# Quantifying the fluxes of carbon loss from an undrained tropical peatland ecosystem in Indonesia

**DOI:** 10.1038/s41598-024-62233-6

**Published:** 2024-05-20

**Authors:** Adibtya Asyhari, Adi Gangga, Chandra Agung Septiadi Putra, Rasis Putra Ritonga, Randi Ade Candra, Gusti Z. Anshari, Jennifer C. Bowen, Clarice R. Perryman, Nisa Novita

**Affiliations:** 1 Yayasan Konservasi Alam Nusantara, Jakarta, Indonesia; 2 Magister of Environmental Science, Tanjungpura University, Pontianak, Indonesia; 3 Department of Soil Science, Tanjungpura University, Pontianak, Indonesia; 4 Department of Earth System Science, Stanford University, Stanford, CA USA

**Keywords:** Biogeochemistry, Climate sciences

## Abstract

Conservation of undrained tropical peatland ecosystems is critical for climate change mitigation as they store a tremendous amount of soil carbon that is preserved under anoxic water-logged conditions. Unfortunately, there are too few measurements of carbon fluxes from these ecosystems to estimate the climate change mitigation potential from such conservation efforts. Here, we measured carbon dioxide (CO_2_) and methane (CH_4_) fluxes as well as fluvial organic carbon export over the peat swamp forest within an undrained tropical peatland landscape in East Kalimantan, Indonesia. Our measurements throughout one year (Oct 2022–Sep 2023) showed that despite its water-logged condition, peat and water overlying the swamp forest on average emits 11.02 ± 0.49 MgCO_2_ ha^−1^ yr^−1^ of CO_2_ and 0.58 ± 0.04 MgCO_2_e ha^−1^ yr^−1^ of CH_4_. Further, the fluvial organic carbon export contributes to additional carbon loss of 1.68 ± 0.06 MgCO_2_e ha^−1^ yr^−1^. Our results help improve the accuracy of carbon accounting from undrained tropical peatlands, where we estimated a total carbon loss of 13.28 ± 0.50 MgCO_2_e ha^−1^ yr^−1^. Nevertheless, the total carbon loss reported from our sites is about half than what is reported from the drained peatland landscapes in the region and resulted in a larger onsite carbon sink potential estimate compared to other undrained peat swamp forests. Together, these findings indicate that conserving the remaining undrained peatland ecosystems in Indonesia from drainage and degradation is a promising natural climate solution strategy that avoids significant carbon emissions.

## Introduction

Tropical peatland ecosystems play important roles in providing a broad array of ecosystem services, including livelihood supplies, biodiversity hotspots, flood control, as well as climate regulation through carbon sequestration^1,2^. Globally, tropical peatland have accumulated at least 75 Gt of carbon due to anoxic water-logged conditions that slow down decomposition processes^3^. Peatland geomorphology, ecology, and biogeochemical cycling are strongly controlled by hydrology^4,5^. Therefore, alterations in the hydrological processes due to climate and land cover changes may have crucial implications for tropical peatland ecosystems. More frequent droughts and drainage for development in peatland areas resulted in notable groundwater level drawdown in tropical peatland ecosystems^6–8^ and thus, may reverse the natural role of tropical peatland ecosystems from carbon sink to carbon source.

Southeast Asia is home to roughly a third of tropical peatlands extent^9^, with Indonesia alone containing approximately 15% of all tropical peatland areas (13.4 million ha)^10^. About half of peatlands in this region are currently degraded and under serious threats due to extensive peatland drainage and land use change that have caused significant carbon emissions^11–14^. However, a recent study^15^ concluded that tropical peatlands in Indonesia have the potential to contribute up to 74% of the total maximum national mitigation potential from natural climate solutions through both conservation and restoration efforts. Therefore, conserving and restoring tropical peatland ecosystems in Indonesia is pivotal for climate change mitigation and thus fulfilling national climate commitments. Further, Indonesia has acknowledged the importance of peatland conservation as part of the country’s transition to a low carbon and climate resilience future, which was stated on Indonesia’s national agenda, such as the Forest and Other Land Use (FOLU) Net Sink or Enhanced Nationally Determined Contribution (ENDC) target^16^.

At the current state, much less is known about the climate change mitigation potential for conserving undrained tropical peatlands in Indonesia. Fewer studies have measured greenhouse gas (GHG) fluxes from undrained tropical peatlands compared to drained peatland landscapes^17–21^, in part because the remaining natural tropical peatland landscape with water-logged condition, if any, is difficult to access. Due to limited data, the current emission factors from Intergovernmental Panel on Climate Change (IPCC) that use both carbon input and loss terms assume that the GHG emissions from undrained tropical peat swamp forests are negligible^22^ because of the absence of anthropogenic disturbances. However, the data that are available demonstrate that undrained tropical peat swamp forests may emit carbon dioxide (CO_2_) to the atmosphere^17–21^ and have substantial methane (CH_4_) emissions from peat and trees^23^. Given that CH_4_ is a more potent GHG compared to CO_2_, this could lead to a substantially lower climate change mitigation potential compared to current estimates.

Further, additional carbon may naturally be released from undrained tropical peatlands through fluvial carbon export to water body. The carbon stored in peat is continuously exported from peatlands through fluvial pathways as dissolved CO_2_, dissolved CH_4_, dissolved organic carbon (DOC), and particulate organic carbon (POC). Once CO_2_ and CH_4_ reach the water body, they may be released into the atmosphere^24,25^. Although fluvial carbon export is generally less understood compared to direct atmospheric GHG fluxes coming from the peat surface, recent research suggests that fluvial carbon export can represent a substantial fraction of the total carbon emission^26–28^.

**Figure 1:**
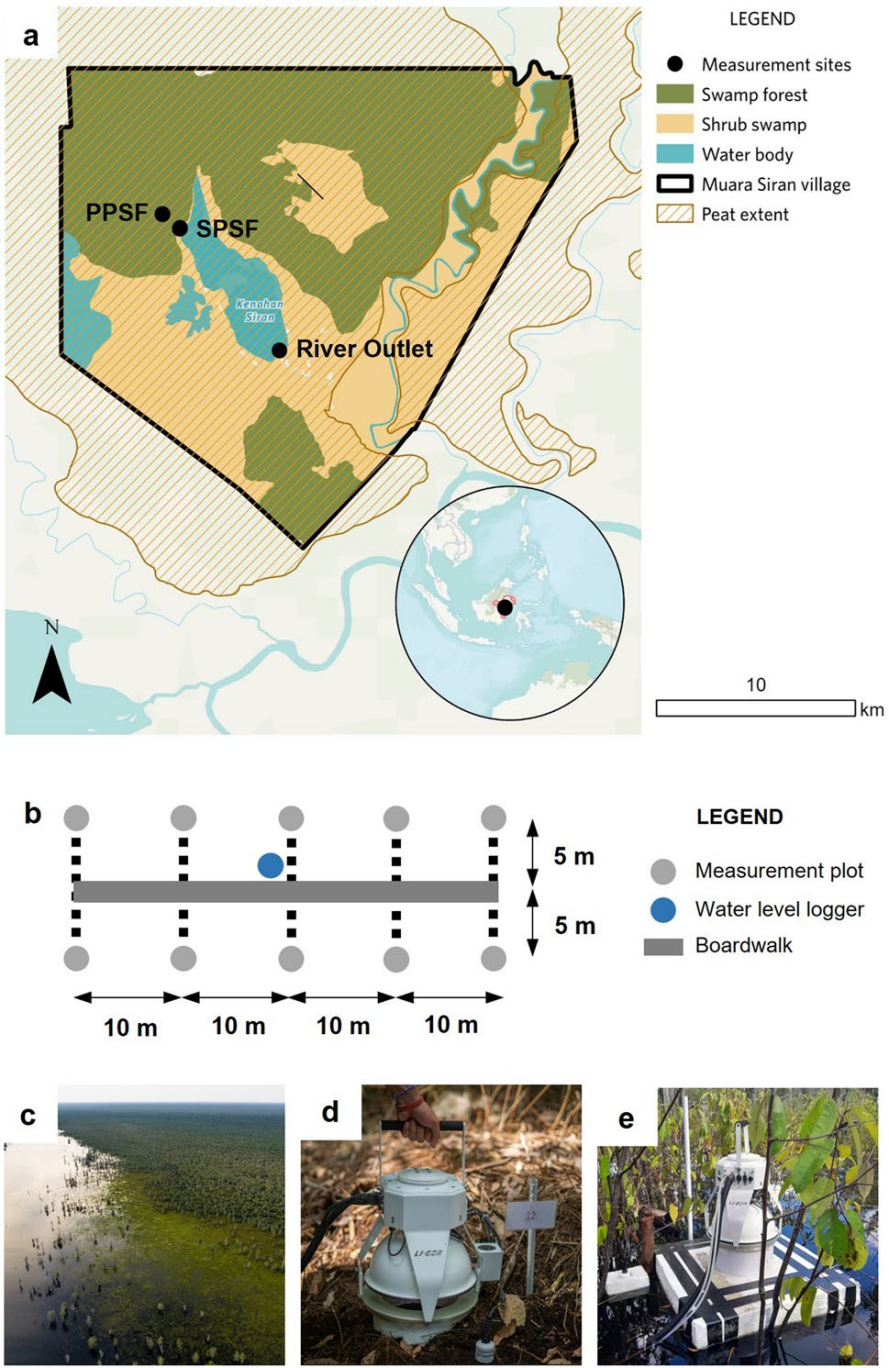
Location of the study area and plot design. (**a**) Land cover map of Muara Siran Village overlaid with the peatland extent from ref^10^. The location of Muara Siran Village in East Kalimantan, Indonesia, is presented in the inset. (**b**) Plot design for peat CO_2_ and CH_4_ fluxes measurement. The aerial view of the study area (**c**) as well as the measurement during dry (**d**) and wet conditions (**e**).

In this study, we aim to quantify the fluxes of carbon loss from an undrained tropical peatland landscape in Muara Siran, East Kalimantan, Indonesia (Fig. 1a). The carbon loss from the peatland was quantified as the CO_2_ and CH_4_ emitted from the peat surface as well as the fluvial carbon export. Environmental variables were measured alongside the flux measurements to evaluate controls on the loss of carbon. The measurements were conducted over two undrained peat swamp forest sites with different levels of disturbance, namely pristine peat swamp forest (PPSF) and secondary peat swamp forest with historical logging (SPSF) (Table 1). To our understanding, this is the first comprehensive study to measure both the vertical and horizontal pathways of carbon loss from undrained tropical peatland landscapes with water-logged condition. The findings of this study can be used to refine the accounting for the climate change mitigation potential of conserving undrained tropical peatland ecosystems.

**Table 1:** Characteristics of the study sites (average ± standard error).

	Site 1 (PPSF)	Site 2 (SPSF)
Description	Pristine peat swamp forest	Secondary peat swamp forest with historical logging
Coordinate	116.5501 E; 0.0333 S	116.5527 E; 0.0354 S
Surface peat classification	Fibric	Fibric
Peat depth (cm)	765 ± 26.6 (n = 6)	730 ± 34.2 (n = 6)
Peat age at 0–5 cm depth (year BP)	100 ± 1 (n = 3)	N/A
Peat age at 720–725 cm depth (year BP)	6790 ± 15 (n = 3)	N/A
Bulk density (g cm^−3^)	0.07 ± 0.00 (n = 5)	0.09 ± 0.00 (n = 5)
Porosity (%)	95.67 ± 0.30 (n = 5)	95.66 ± 0.27 (n = 5)
Total organic carbon (%)	50.63 ± 0.94 (n = 54)	50.12 ± 0.78 (n = 48)
Total nitrogen (%)	1.12 ± 0.03 (n = 54)	1.14 ± 0.02 (n = 48)
pH	3.35 ± 0.05 (n = 5)	3.53 ± 0.07 (n = 5)
Aboveground carbon stock (MgC ha^−1^)	231.2 ± 50.9 (n = 6)	201.9 ± 53.5 (n = 6)
Belowground carbon stock (MgC ha^−1^)	2883.7 ± 212.8 (n = 6)	3489.7 ± 116.2 (n = 6)

## Results

### Peat properties and hydrology of the area

We found that the properties of peat underlying both PPSF and SPSF were similar (Table 1). The peats in both sites were classified as fibric and characterized by high carbon content, acidic pH, low bulk density, and high porosity. The radiocarbon age of the peat in PPSF indicates that the surface peat layer (0–5 cm) was about 100-year BP, while the basal peat layer (720–725 cm) was formed 6790 year BP. The aboveground carbon stock in PPSF was similar to that in SPSF (231.2 ± 50.9 and 201.9 ± 53.5 MgC ha^−1^, respectively; p = 0.16), while the belowground carbon stock in PPSF was lower than that in SPSF (2833.7 ± 212.8 and 3489.7 ± 116.2 MgC ha-1, respectively; p < 0.05).

**Figure 2:**
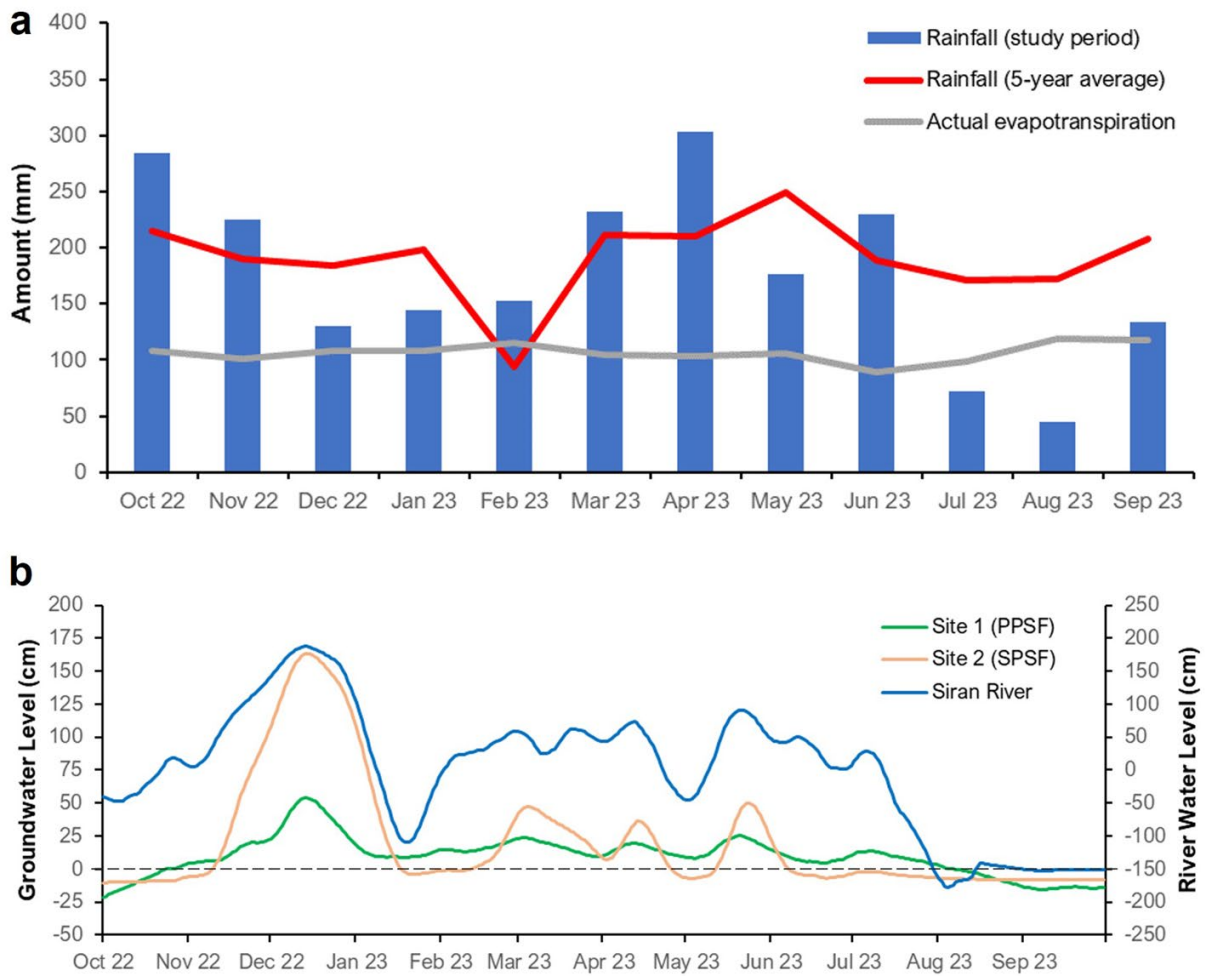
Temporal variability of rainfall and water level across our study sites within the study period. (**a**) The rainfall observed in the study period overlaid with the five-year average rainfall data (2018–2022) obtained from Samarinda meteorological station in East Kalimantan, Indonesia and actual evapotranspiration calculated using the FAO Penman–Monteith method. (**b**) The groundwater level data from automatic water level loggers across our two measurement sites overlaid with river water level in the Siran River.

Our study period can be considered representative of a normal year as the annual rainfall during the study period was similar to the five-year average across 2018–2022 (2130 mm vs 2292 mm, respectively) (Fig. 2a). The annual actual evapotranspiration in the study area during the study period was 1282 mm. Assuming that the difference between annual rainfall and actual evapotranspiration equaled runoff (i.e. no change in water storage within the peatland ecosystem), annual runoff during the study period was approximately 848 mm.

The groundwater level of both PPSF and SPSF and the water level of Siran River fluctuated throughout the year following changes in rainfall (Fig. 2a,b). For example, the groundwater and river water level had peaks in December 2022 and June 2023 following periods of high rainfall, while following periods of lower rainfall, water levels declined in January 2023 and July 2023. Within the plots where CO_2_ and CH_4_ fluxes were measured, we observed more fluctuating groundwater levels in SPSF compared to PPSF (Fig. 2b), consistent with the closer proximity of the SPSF plots to the river (Fig. 1a). Overall, the groundwater level in PPSF (19.01 ± 0.80 cm above the peat surface) was lower than that in SPSF (37.34 ± 1.91 cm above the peat surface; p < 0.05) (Table 2). We also observed a staggering ~ 4 m difference between the highest and lowest water levels in the Siran River (Fig. 2b).

**Table 2:** Summary of CO_2_ and CH_4_ fluxes across our study sites (average ± standard error).

	Site 1 (PPSF)	Site 2 (SPSF)
Groundwater level (cm)	19.01 ± 0.80 (n = 360)	37.34 ± 1.91 (n = 360)
CO_2_ flux (MgCO_2_ ha^−1^ yr^−1^)	12.61 ± 0.41 (n = 360)	9.43 ± 0.27 (n = 360)
CH_4_ flux (MgCO_2_e ha^−1^ yr^−1^)	0.70 ± 0.04 (n = 360)	0.46 ± 0.02 (n = 360)
Total of CO_2_ and CH_4_ fluxes (MgCO_2_e ha^−1^ yr^−1^)	13.31 ± 0.41	9.89 ± 0.27

### Carbon Dioxide and Methane Fluxes

**Figure 3:**
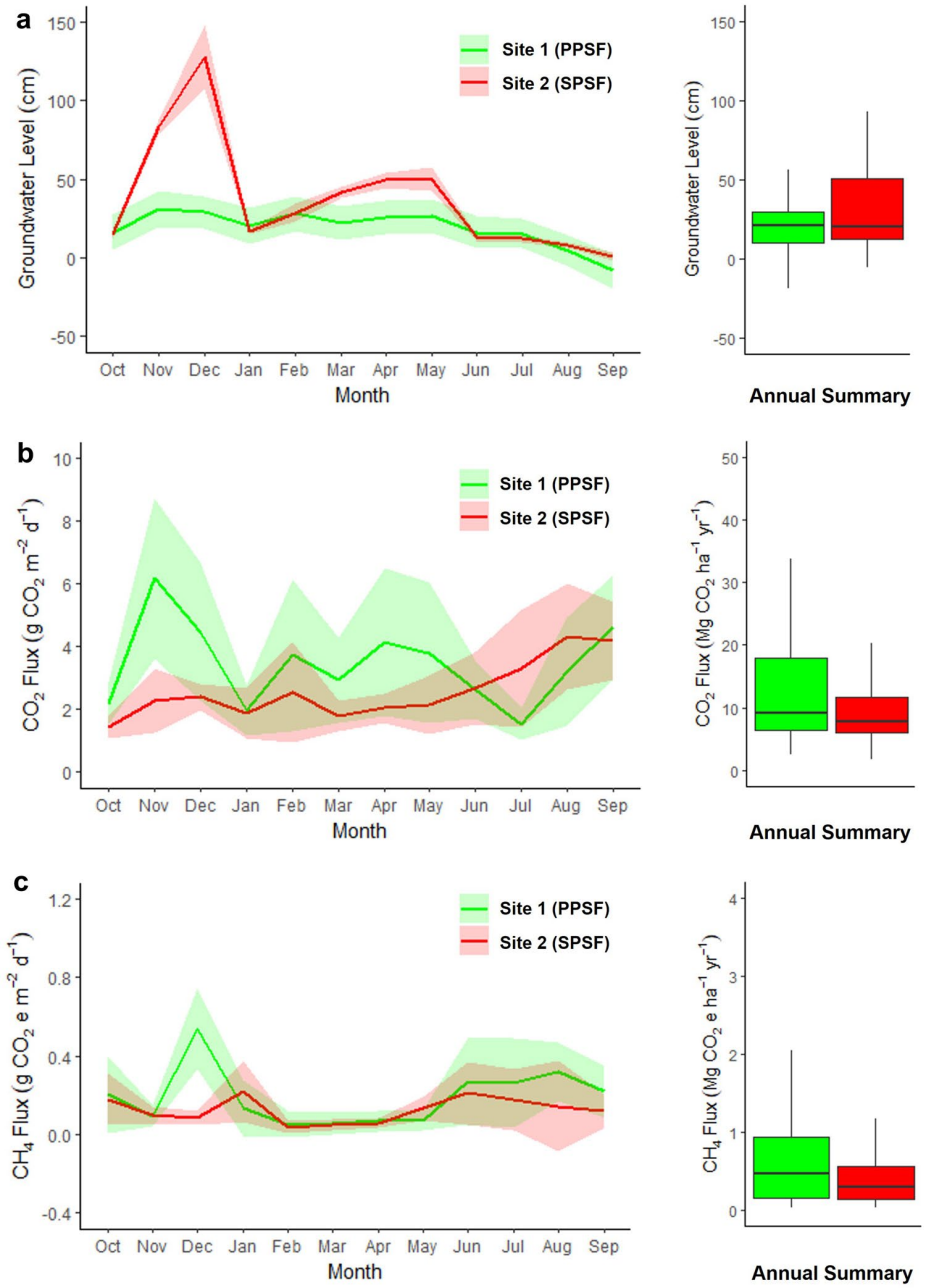
Monthly variability of groundwater level and GHG fluxes across our study sites. The panels include (**a**) groundwater level measurement conducted manually at the same time as the (**b**) CO_2_ fluxes, and (**c**) CH_4_ fluxes measurements. The lines connect the average monthly data, while the shaded areas indicate the standard error of the measurements (n = 30 measurements made each month).

**Figure 4:**
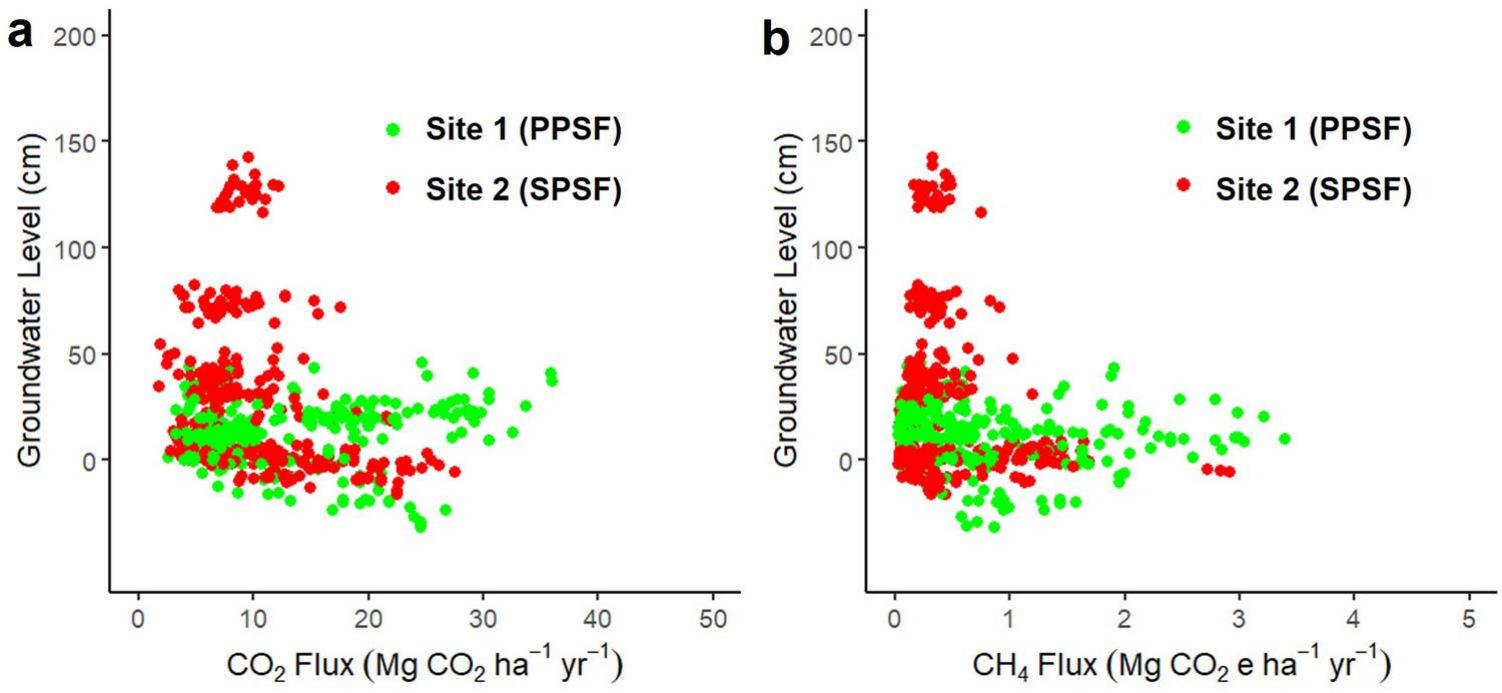
Relationship between (**a**) CO_2_ and (**b**) CH_4_ fluxes and groundwater level at both PPSF and DPSF sites. Each scatterplot shows 360 measurements from each site.

The fluctuation of groundwater level influenced CO_2_ and CH_4_ fluxes throughout the year (Fig. 3a–c). However, we did not find a strong relationship between both CO_2_ and CH_4_ fluxes and groundwater level (Fig. 4a,b). The CO_2_ and CH_4_ fluxes varied more widely for SPSF compared to PPSF following the larger changes in groundwater level observed for SPSF. The lower average groundwater level in PPSF coincided with the highest observed CO_2_ and CH_4_ fluxes (0–40 cm above the peat surface; Fig. 4a,b). In contrast, when groundwater levels were higher in SPSF, some of the lowest CO_2_ and CH_4_ fluxes were observed (Fig. 4a,b). As a result, on a monthly basis, the CO_2_ and CH_4_ fluxes from PPSF were generally larger than those from SPSF (Fig. 3b,c).

On an annual basis, the peats and waters overlying from both PPSF and SPSF emit carbon to the atmosphere, with 13.31 ± 0.41 and 9.89 ± 0.27 MgCO_2_e ha^-1^ yr^-1^ respectively (Table 2). PPSF emitted significantly larger annual CO_2_ and CH_4_ fluxes (12.61 ± 0.41 MgCO_2_ ha^-1^ yr^-1^ of CO_2_ and 0.70 ± 0.04 MgCO_2_e ha^−1^ yr^−1^ of CH_4_) compared to SPSF (9.43 ± 0.27 MgCO_2_ ha^-1^ yr^-1^ of CO_2_ and 0.46 ± 0.02 MgCO_2_e ha^−1^ yr^−1^ of CH_4_; p < 0.05). For both PPSF and SPSF, CH_4_ fluxes represented about 5% of the total carbon flux.

### Fluvial carbon export

**Figure 5:**
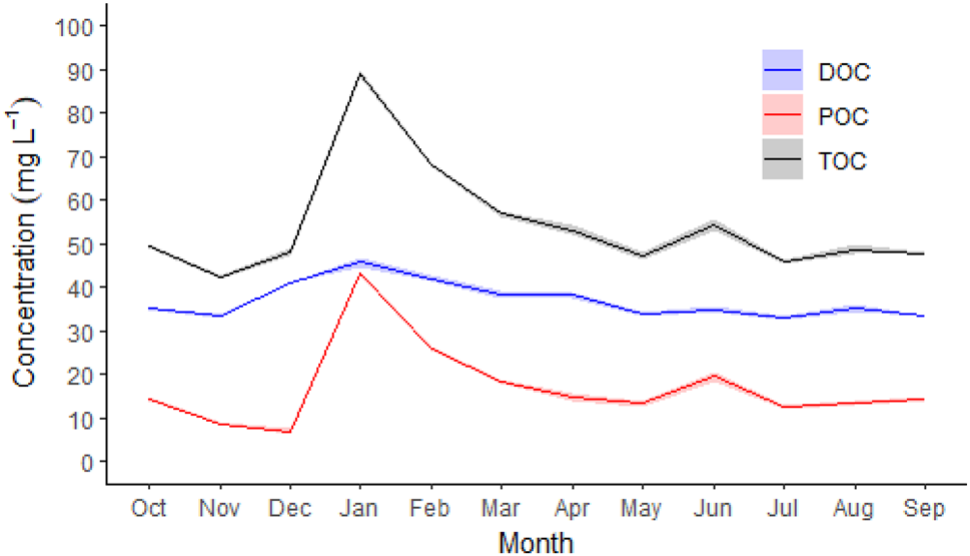
Monthly variability of dissolved organic carbon (DOC), particulate organic carbon (POC), and total organic carbon (TOC) concentrations. The lines connect the average monthly data, while the shaded areas indicate the standard error of the triplicate field measurements (n = 3).

The concentration of total organic carbon (TOC, calculated as sum of DOC and POC) in Siran River increased following the flood period, as observed with the highest TOC concentration in January 2023 (Fig. 5). The TOC concentrations were more affected by the fluctuations in POC concentration, as there was no substantial fluctuation in DOC concentration, which remained relatively stable for the duration of the study. Overall, the average annual TOC concentration in Siran River was 54.20 ± 2.07 mg L^−1^ (Table 3). Here, we found that DOC was the dominant organic component accounting for 68% of the TOC. Considering the annual runoff, fluvial carbon export from the study area was 1.68 ± 0.06 MgCO_2_e ha^−1^ yr^−1^.

**Table 3:** Summary of fluvial carbon export across our study sites (average ± standard error).

	Muara Siran
Water pH	4.29 ± 0.07 (n = 36)
Water temperature (^o^C)	30.2 ± 0.25 (n = 36)
DOC concentration (mg L^−1^)	37.10 ± 0.66 (n = 36)
POC concentration (mg L^−1^)	17.20 ± 1.55 (n = 36)
TOC concentration (mg L^−1^)	54.20 ± 2.07
Fluvial carbon export (MgCO_2_e ha^−1^ yr^−1^)	1.68 ± 0.06

## Discussion

In this study, we quantified the carbon loss via CO_2_ and CH_4_ fluxes from the peat or the inundated water surface during flood period as well as fluvial organic carbon export from undrained tropical peat swamp forests. Our findings show that by integrating these three fluxes of carbon loss across the two sites, the Muara Siran peatland ecosystem has an annual carbon loss of 13.28 ± 0.50 MgCO_2_e ha^-1^ yr^-1^. The majority of carbon is lost as CO_2_ flux (11.02 ± 0.49 MgCO_2_ ha^−1^ yr^−1^), followed by fluvial organic carbon export (1.68 ± 0.06 MgCO_2_e ha^−1^ yr^−1^) and CH_4_ flux (0.58 ± 0.04 MgCO_2_e ha^-1^ yr^−1^). Compared to past studies of carbon fluxes from both drained and undrained tropical peatlands^17–21^, we observed substantially lower CO_2_ fluxes (Table 4). The lower CO_2_ fluxes observed at our site compared to other undrained tropical peatlands is likely due to the high groundwater levels, which across both PPSF and SPSF sites are the highest reported for an undrained peatland to date in the region^18–21^. Meanwhile, the annual CH_4_ emissions from both PPSF and SPSF were comparable to those previously reported from peat swamp forests with lower annual groundwater levels^17–21^.

**Table 4:** Comparison of CO_2_ and CH_4_ fluxes reported across tropical peatlands (average ± standard error).

Land cover	Average groundwater level (cm)	CO_2_ Flux (MgCO_2_ ha^−1^ yr^−1^	CH_4_ Flux (MgCO_2_e ha^−1^ yr^−1^)
This study			
Undrained undegraded peat swamp forest (PPSF)	19.01	12.61 ± 0.41	0.70 ± 0.04
Undrained secondary peat swamp forest with historical logging (SPSF)	37.34	9.43 ± 0.27	0.46 ± 0.02
IPCC default emission factor^22^			
Undrained peat swamp forest^22^	N/A	0.00	1.10
Drained peat swamp forest^22^	N/A	19.45	0.13
Drained oil palm plantation^22^	N/A	40.37	0.00
Drained acacia plantation^22^	N/A	73.40	0.07
Synthesis of Southeast Asian Peatlands^36^			
Undrained undegraded peat swamp forest^36^	− 31.3 ± 4.5	38.1 ± 6.6	0.80 ± 0.49
Undrained degraded peat swamp forest^36^	− 14.8 ± 3.9	55.3 ± 7.3	1.18 ± 0.28
Other studies			
Undrained peat swamp forest with historical logging^18^	− 20.00	38.92 ± 3.04	0.18 ± 0.08
Undrained peat swamp forest^19^	N/A	23.46 ± 0.45	0.38 ± 0.11
	N/A	26.34 ± 0.91	0.46 ± 0.12
	N/A	27.97 ± 0.97	0.47 ± 0.13
Undrained peat swamp forest^20^	1.00	33.29 ± 7.12	1.61 ± 0.45
	− 1.00	37.87 ± 8.61	1.49 ± 0.91
Undrained peat swamp forest^21^	− 12.60	43.63 ± 1.83	0.70 ± 0.06
	− 7.30	42.21 ± 1.80	1.67 ± 0.11
	− 3.90	46.68 ± 1.86	2.97 ± 0.22
Drained peat swamp forest^17^	− 10.00	44.04 ± 9.11	0.43 ± 0.14
Drained peat swamp forest with historical logging^19^	N/A	37.19 ± 3.83	0.30 ± 0.07
	N/A	30.21 ± 3.56	0.34 ± 0.08

The negative groundwater level indicates that the value was below the peat surface.

We also compared the CO_2_ and CH_4_ fluxes between PPSF and SPSF, which represent pristine and historically logged portions of the undrained peatland, respectively. Here, we found that PPSF had a larger estimate of carbon loss (Table 2), which was driven by higher fluxes of CO_2_ compared to SPSF. The higher CO_2_ fluxes from the PPSF may be due to the lower annual groundwater level at this site (Table 2) as heterotrophic respiration increases with lower groundwater level^29^. Further, the relatively higher tree density in PPSF compared to SPSF could have led to greater autotrophic respiration, a factor that was not parsed out from our total CO_2_ flux estimates. While surface vegetation was removed in our plots prior to any CO_2_ and CH_4_ fluxes measurements, any belowground root mass would have remained intact within the peat. If there were larger belowground root masses within PPSF compared to SPSF, this could have impacted CO_2_ fluxes because root respiration has been shown to contribute about 25–50% of total peat respiration in tropical peatland^30^.

The larger annual CH_4_ fluxes from PPSF were also likely due to lower groundwater levels than SPSF (Table 2). Methane emissions from undrained tropical peatlands have been observed to peak when groundwater levels are about 5 cm above the peat surface and decline as groundwater levels rise above this level^20^. High groundwater levels may prevent CH_4_ fluxes by increasing hydrostatic pressure. High groundwater levels can also enhance opportunities for CH_4_ oxidation in the overlying water column, decreasing CH_4_ fluxes from the water surface. Consistent with these explanations, in SPSF, we observed an increase in CH_4_ fluxes as the groundwater level fell from its maximum level of > 1 m in December 2022 to near the peat surface in January 2023 (Fig. 3a,c). A similar phenomenon has also been observed in undrained tropical peatlands in South America where low CH_4_ fluxes were observed alongside high groundwater levels during the wet season^31^.

Past efforts to quantify carbon fluxes from either undrained and drained peatland have not yet combined both vertical and horizontal pathways. Our estimates of annual TOC export from the Muara Siran peat swamp suggest that an additional ~ 13% carbon may be lost from undrained tropical peatlands through fluvial pathways. The estimated carbon loss through the aquatic system from our site (1.68 ± 0.06 MgCO_2_e ha^−1^ yr^−1^) is within the values previously reported for other peatlands across Southeast Asia (0.96–3.86 MgCO_2_e ha^−1^ yr^−1^)^26,28^. Our estimate of TOC export is also comparable with the current emission factors adopted by IPCC of 2.10 MgCO_2_e ha^−1^ yr^−1^^22^. The enhanced TOC export observed following the raised groundwater level in Muara Siran suggests that fluvial exports from peatland ecosystems dominate most TOC exports from Siran River. Further, litter and woody debris export from the peat swamp forests during peak flow could have led to increased POC concentrations within the TOC pool. Our findings demonstrate that the fluvial pathways contribute a prominent portion of the total carbon export from the undrained tropical peat swamp forests and should be considered in carbon accounting.

Critically, our results demonstrate that when taken together, CO_2_ and CH_4_ fluxes as well as fluvial organic carbon export from our study site are about half of those reported from drained tropical peat swamp forests in the region (Table 4). These findings highlight that conserving undrained tropical peatland ecosystems can avoid substantial carbon emission caused by peatland drainage. However, areas of uncertainty in our flux estimates may lead to higher or lower estimates of the total emissions avoided through tropical peatland conservation. For example, our estimates of the CO_2_ flux from the peat surface may be overestimated because we cannot rule out the contribution of autotrophic respiration to the total CO_2_ flux measured. Past estimates suggest that the contribution of heterotrophic respiration to the total peat CO_2_ flux in intact peat swamp forests in Southeast Asia is 54%^32,33^ while the percentage can go up to 81% in degraded undrained peat swamp forests^20,34^. Our estimates of carbon loss through fluvial export may also be overestimated because we do not know the portion of TOC that will get oxidized to CO_2_ and emitted to the atmosphere in transit from the peatland soil to the Siran River. Recent studies estimated that the majority of the DOC exported from Indonesian peatland soils may undergo oxidation to CO_2_ in freshwaters^35^ and the coastal sea^36^, but the susceptibility of POC to oxidation remains unknown. On the other hand, our estimates of CH_4_ fluxes may be underestimated. Tree-mediated CH_4_ emissions can be substantial in tropical peatlands, accounting for 60–80% of total ecosystem CH_4_ fluxes^23^. Recent estimates of CH_4_ emissions from rivers also suggest that tropical rivers in peatland dominated catchments can have substantial CH_4_ emissions^37^. As we do not account for tree-mediated emissions nor any potential CH_4_ degassing from the Siran River, our estimates of CH_4_ emissions from Muara Siran should be considered as a minimum annual value.

Despite these areas of uncertainty, the fluxes of carbon loss quantified in our study may improve estimates for the potential onsite CO_2_ emissions of undrained peat swamp forests. Estimating onsite peat CO_2_ emissions following IPCC guidelines requires values for both carbon loss via heterotrophic respiration and the carbon input from litterfall and root mortality. Applying literature values for carbon inputs and soil respiration partitioning ratios specific to peatlands in Southeast Asia^38^, we estimate that the peat onsite CO_2_ budget for the PPSF is − 25.1 ± 7.5 MgCO_2_e ha^−1^ yr^−1^ and for the SPSF is – 22.0 ± 5.6 MgCO_2_e ha^−1^ yr^−1^ (Table 5). These estimates for the potential onsite CO_2_ sink for the PPSF and SPSF are much larger than those reported for undegraded and degraded undrained peatlands in Southeast Asia^38^. Our higher estimates for the onsite CO_2_ sink potential may be due to the lower estimated heterotrophic respiration rates at Muara Siran compared to other undrained peatlands. We attribute this difference to the year-round water-logged conditions at Muara Siran that limit peat oxidation and therefore heterotrophic respiration. Extending our calculations to account for carbon losses via CH_4_ fluxes and fluvial carbon export, we estimate the carbon budget of the PPSF is − 22.7 ± 7.4 MgCO_2_e ha^−1^ yr^−1^ and of the SPSF is − 19.9 ± 5.6 MgCO_2_e ha^−1^ yr^−1^. These estimates show the potentially large carbon sink potential of Muara Siran peatland and other remaining undrained peatlands in Southeast Asia.

**Table 5:** Estimation of peat swamp forest onsite CO_2_ budget and net carbon budget (average ± standard error) using the synthesis data from peatlands across Southeast Asia (SEA)^38^.

	Undegraded Undrained		Degraded Undrained	
	SEA^38^	PPSF (This study)	SEA^38^	SPSF (This study)
Litterfall (MgC ha^−1^ yr^−1^)	6.7 ± 0.6	–	6.9 ± 0.6	–
Root mortality (MgC ha^−1^ yr^−1^)	2.0 ± 1.5	–	1.2 ± 1.0	–
Total soil respiration (MgC ha^−1^ yr^−1^)	10.4 ± 1.8	3.4 ± 0.1	15.1 ± 2.0	2.6 ± 0.1
Heterotrophic respiration (MgC ha^−1^ yr^−1^)	5.7 ± 1.0	1.9 ± 0.1	12.2 ± 1.6	2.1 ± 0.1
**Onsite CO**_**2**_** budget (MgC ha**^**−1**^** yr**^**−1**^**)**	** − 2.9 ± 1.8**	** − 6.8 ± 2.0**	**4.1 ± 2.0**	** − 6.0 ± 1.5**
**Onsite CO**_**2**_** budget (Mg CO**_**2**_**e ha**^**−1**^** yr**^**−1**^**)**	** − 10.8 ± 6.7**	** − 25.1 ± 7.5**	**15.0 ± 7.2**	** − 22.0 ± 5.6**
Additional carbon losses				
Methane flux (MgCO_2_e ha^−1^ yr^−1^)	–	0.70 ± 0.04	–	0.46 ± 0.02
Fluvial export (MgCO_2_e ha^-1^ yr^−1^)	–	1.68 ± 0.06	–	1.68 ± 0.06
**Peat carbon budget (MgCO**_**2**_**e ha**^**−1**^** yr**^**−1**^**)**	**–**	** − 22.7 ± 7.4**	**–**	** − 19.9 ± 5.6**

The onsite CO_2_ budgets were calculated as the difference of mean annual C outputs from heterotrophic soil respiration and mean annual C inputs from litterfall and root mortality. The peat C budgets were calculated from the onsite CO_2_ budget considering the additional C loss from CH_4_ emission and fluvial carbon export. For this study, the heterotrophic respirations were partitioned from total CO_2_ flux using a ratio of 0.54 for PPSF^32^ and 0.81 for SPSF^20^. The fluvial carbon flux coming from each peatland site (PPSF versus SPSF) was assumed to be equal. Negative values indicate an emission reduction or removal.

The aggregate values are in bold.

In the context of climate change mitigation, the Government of Indonesia (GoI) is determined to fulfill its climate commitment as stated in the ENDC. Conservation, management, and restoration efforts within the FOLU sector is expected to contribute up to 55% of the total emissions reduction target for Indonesia^16^. Within these plans, the GoI has committed to halting and reversing forest loss and degradation of peatlands to achieve Indonesia’s FOLU Net Sink by 2030. Based on the notion of that *“we cannot manage what we cannot measure”*, more estimates of carbon loss are needed from undrained tropical peatlands to provide the robust carbon accounting required to fulfill Indonesia’s climate commitment. Further, robust estimation of carbon accounting can also support the implementation of carbon market regulation by providing appropriate baselines and measuring emission reduction performance. The findings from this study help inform future IPCC methodology for national GHG inventories by providing emissions factors for an undrained tropical peatland ecosystem that was not previously included in the IPCC guidelines^22^. Nevertheless, long-term measurements of carbon inputs and losses from our site and other undrained tropical peatland ecosystems are required to understand whether the large carbon sink potential in Muara Siran peatland ecosystem is site- and time-specific or whether these findings are representative of the other remaining undrained tropical peatland ecosystems in Indonesia.

The constraints in time and resources remain the biggest hurdles to mitigating climate change. Our findings show that conserving the Muara Siran peatland landscape and other similar ecosystems with high groundwater levels in Indonesia may serve as a promising natural climate solution. Following the natural climate solutions hierarchy of protect, manage, and restore^39^, protection of the remaining natural peatland landscape should be prioritized as such effort is more cost-effective and has a greater magnitude and immediate climate mitigation benefit, as well as offer co-benefits beyond climate change mitigation.

## Methods

### Study area

This study was conducted in Muara Siran Village (ca. 42,200 ha), that is situated on the bank of Mahakam River, in the East Kalimantan province of Indonesia (Fig. 1a). The land cover in the study area is dominated by swamp forest and shrub swamp, with a notable presence of water body in the form of Siran Lake in the middle of the area. The Siran River flows south of the lake to join Kedang Kepala River downstream of the village and functions as an outlet for the upper catchments.

Human activities and fires are the most prominent threats to the peatland ecosystem in Muara Siran. Although the peatland ecosystem in Muara Siran can be considered as relatively intact, they have faced logging and fires in the past. Slash and burn practices used to be a common way to clear the aboveground vegetation during the dry period, and this has made the land vulnerable to peat fires^40^. Currently, the economy in the area is centered around swiftlets business and fisheries.

The area has a humid tropical climate with average air temperature ranging between 25 to 30 °C. The interannual variability of rainfall is influenced by the El Niño Southern Oscillation and the Indian Ocean Dipole^41^. The average annual rainfall for the five-year period prior to field measurements (2018–2022) was 2292 ± 210 mm (average ± standard error) and characterized by high seasonal variability (Fig. 2). There are frequent flood episodes throughout the year. During the flood episode, prolonged inundation can be expected for up to three months.

### Measurement of CO_2_ and CH_4_ Fluxes

Fluxes of CO_2_ and CH_4_ from the peat and inundated water during flood period were measured from two peat swamp forest sites with different levels of disturbance, namely pristine peat swamp forest (PPSF) and secondary peat swamp forest with historical logging (SPSF). For each site, ten measurement plots were established in a 2 by 5 grid with the plots spaced 10 m apart (Fig. 1b). Here, our sampling plots were designed to capture the spatial heterogeneity of CO_2_ and CH_4_ fluxes and environmental conditions by considering microtopography and vegetation cover within each site (Fig. 1c). The SPSF plots were situated closer to the Siran River compared to the PPSF plots, which were further upslope **(**Fig. 1a). In this study, the plot establishment was conducted about a month prior to the first measurement to avoid potential influence from disturbance during plot installation. During the plot establishment, we installed 8-inch PVC collar by inserting about 5 cm of the PVC collar into the peat surface until it was sealed to the ground surface. Upon installation, the surface aboveground vegetation within the PVC collar was cleared. In addition, a 0.5-inch PVC pole was installed at each measurement plot to be used for measuring groundwater level and designating the plot location during flooded periods when the PVC collar was inundated.

The fluxes were measured using a LICOR LI-7810 portable GHG analyzer (LICOR Biosciences, USA). The measurements were conducted following a closed chamber technique using LICOR 8200-01S smart chamber (LICOR Biosciences, USA) equipped with a Stevens HydraProbe^®^ (Stevens Water, USA) consisting of soil moisture, soil temperature, and electrical conductivity probes. For each measurement, we put the LICOR 8200-01S smart chamber on top of the 8-inch PVC collar (Fig. 1d). During the flood season, we utilized a different technique to measure peat GHG fluxes, in which we developed a floating chamber device made of Styrofoam and 8-inch PVC collar (Fig. 1e).

The CO_2_ and CH_4_ flux measurements were conducted on a monthly basis for a period of one year, from October 2022 to September 2023. For each monthly measurement, the ten plots were sampled once per day within a specific time window (0800 h to 1200 h) for three consecutive days to capture the range of possible fluxes taking place within the month. During the measurement at each plot, the LICOR 8200-01S smart chamber was placed over the PVC collar for a total of 2 min while CO_2_ and CH_4_ concentrations were being measured. This was repeated two additional times to obtain 3 experimental replicate measurements per day. Upon data collection in the field, SoilFluxPro software was used to compute the fluxes. Here, the linear fit was selected by specifying a dead band of 10 s and stop time of 120 second^42^. The flux measurements were quality filtered to include only fluxes with sufficient linear fits with a coefficient of determination higher than 0.9 and 0.7 for CO_2_ and CH_4_ fluxes, respectively. The CH_4_ fluxes are then expressed as CO_2_e by multiplying the amount of CH_4_ fluxes with 27 as Global Warming Potential (GWP) over a 100 years period^43^. The annual CO_2_ and CH_4_ fluxes were calculated from the average of individual fluxes measured across the measurement sites. During the study period, the majority of flux measurements (72%) were made from the water surface of the inundated peat. Hence, we adopt terminology used in the aquatic science community to describe CO_2_ and CH_4_ loss as diffusive fluxes (i.e. CO_2_ and CH_4_ fluxes).

### Measurement of environmental variables

We took the measurements of environmental variables concomitantly with the fluxes measurements. The groundwater level was measured manually within the 0.5-inch PVC dip wells situated at each plot. A DCX-22 automatic water level logger (Keller, USA) was installed at the center of the plot for each site to record the continuous groundwater level data on an hourly interval. In addition, an automatic water level logger was also installed at the outlet of the Siran River to record river water levels throughout the study period.

The above- and belowground carbon stocks at each site were assessed in September 2023 following methodology described in Ref.^44^. For each site, six measurement plots with a radius of 7 m were established 50 m apart along a 250 m transect. These plots were situated about 100 m from the location of flux measurement to ensure that the flux measurements were not affected by the carbon stock assessment.

Peat samples for physical and chemical analysis were also collected in September 2023 from both PPSF and SPSF at 0–50 cm depth using a peat auger. For each site, five samples were taken from the peat in between the two transects of plots established for the CO_2_ and CH_4_ fluxes measurement (Fig. 1b). For soil physical analysis, we determined bulk density and porosity using a gravimetry approach following methodology described in Ref.^13^. The peat was later classified into fibric, humic, or sapric according to von Post humification scale. For chemical analysis, the soil samples were sent to Tanjungpura University, Pontianak, Indonesia for analysis of pH, as well as total carbon and nitrogen content. Here, the total carbon (%) and total nitrogen (%) were measured using Yanaco JM 1000 CN Corder, with hippuric acid as a standard. Additionally, for PPSF, the peat from the top (0–5 cm) and bottom (720–725 cm) of the total peat depth were sampled and prepared for radiocarbon dating analysis following methodology described in Ref.^45^. The samples were shipped to Radiocarbon Dating Laboratory, University of Waikato, Hamilton, New Zealand to measure the radiocarbon isotope composition of peat carbon by Accelerator Mass Spectrometry (AMS). The radiocarbon age of peat was calculated from the radiocarbon isotopic composition as described in Ref.^45^.

The meteorological data, namely rainfall, air temperature, relative humidity, solar radiation, and wind speed, were obtained from a HOBO U30 weather station (ONSET, USA) installed in an open area close to the village office. The instrument provided continuous measurements at 15-min intervals.

### Measurement of fluvial carbon export

Fluvial organic carbon export was estimated from measurements of TOC concentrations at the outlet of the Siran River. In this study, we measured TOC export, which consists of DOC and POC components. Water samples were collected from the Siran River outlet on a monthly basis in parallel with the CO_2_ and CH_4_ fluxes measurement at the peat swamp forests. For each sampling period, six water samples (3 samples for DOC and POC analysis each) were collected within the same day at the same location in pre-rinsed 150 mL amber glass bottles.

DOC samples were filtered in a field laboratory through 0.45 μm cellulose nitrate membrane filters, which were pre-rinsed with water sample, using a vacuum pump within 24 h of collection. After filtration, water samples were stored in the dark at 4 °C to ensure DOC preservation^46^. The filtered samples were sent to Universitas Islam Indonesia, Yogyakarta, Indonesia, for analysis. DOC concentration was determined using a Total Organic Carboniser (Shimadzu, Japan) following the non-purgeable organic carbon method.

POC concentrations were determined gravimetrically following the methodology described in Ref.^47^. The samples were filtered through 0.55 μm glass fibre filters which were then dried for 3 h at 105 °C, weighed, and combusted in a furnace for a further 3 h at 550 °C and reweighed. The particulate organic matter (POM) concentrations were calculated from the difference in the filter mass between oven-drying and combustion divided by the volume filtered. The values were subsequently converted into POC assuming a 50% organic carbon content^48^.

The annual TOC fluxes (J: g C m^−2^ yr^−1^) were calculated from the following formula:

$$J= {C}_{W} x {R}_{E}$$

In which, C_W_ (g C m^-3^) is the mean TOC concentration and R_E_ is the annual run-off (m yr^−1^). The mean annual TOC concentration was calculated as the average of TOC concentrations measured each month (TOC = DOC + POC). Here, calculation of R_E_ from the riverine discharge was not feasible due to seasonal flooding. Thus, R_E_ was estimated using a water balance approach. By assuming no annual change in catchment water storage, R_E_ can be estimated from the climate data using the following formula:

$${R}_{E}= P-{ET}_{a}$$

In which, P is the annual rainfall (m yr^−1^), and ET_a_ is the annual actual evapotranspiration (m yr^−1^), that was calculated from:

$${ET}_{a}={k}_{C} x {ET}_{0}$$

In which, kC is the so-called “crop coefficient” and ET_0_ is the annual reference evapotranspiration rate (mm yr^-1^). ET_0_ was calculated from average daily values of air temperature, humidity, solar radiation, and wind speed using the FAO Penman–Monteith method^49^. In the study area, we assumed that the soil moisture availability will not be a limiting factor and adopted a value of 1 for k_C_ over the whole year. The contribution of the fluvial carbon export to the total carbon loss from the Muara Siran peatland area was calculated as the annual TOC export divided by the sum of annual CO_2_ and CH_4_ fluxes and the annual TOC export.

Alongside the monthly sampling of DOC and POC from Siran River outlet, we took the measurements of the water pH and temperature at a 5 cm depth in the river using HI 9813–6 Portable Water Quality Meter (Hanna Instruments, USA).

### Statistical analysis

The field data were analyzed and visualized using R Statistical Software version 4.2.0. Prior to analysis, we removed data outliers using an objective procedure (i.e. graphical approach) to avoid anomalous measurements, which may be caused by disturbance during field measurement. We used mixed-effects models to assess differences in GHG fluxes and environmental variables between our measurement sites. For each response variable, site and month of measurement were defined as the fixed effects, while the measurement plot number was included as a random effect using the “nlme” package. The level of significance for all analyses was set to be 0.05. We used one-way analysis of variance (ANOVA) to assess the significance of the fixed effects. Further, we conducted post hoc Tukey tests via using the “*emmeans*” package for pairwise comparison.

## Data Availability

All data that support the findings of this study are archived on 10.5281/zenodo.10427000.
